# Research on the relationship between parental media literacy and preschool children’s quality of learning in the new media environment

**DOI:** 10.3389/fpsyg.2025.1600859

**Published:** 2025-11-20

**Authors:** Xiaoyun Zhou, Chen fang Yu, Zhiming Zheng

**Affiliations:** 1 College of Education, Fujian Normal University, Fuzhou, Fujian, China; 2 School of Education, Central China Normal University, Wuhan, China

**Keywords:** parental media literacy, democratic parenting style, screen exposure time, preschool school, learning quality

## Abstract

This study explores the influence of parental media literacy on preschool children’s quality of learning, by focusing on the mediating role of democratic parenting styles in the relationship between the two factors and the group differences of the mediating model in different lengths of screen exposure time. Through a questionnaire-based methodology, the media literacy, parenting style, learning quality, and screen exposure time of 603 parents of preschool children in southern China were acquired. The results show that: (1) After controlling for age and monthly income, 3 media literacy has a significantly positive predictive effect on preschool children’s quality of learning; (2) Democratic parenting can play a mediating role in the relationship between parents’ media literacy and preschool children’s quality of learning; (3) The mediating model of parental media literacy on preschool children’s quality of learning has specific group differences in different lengths of screen exposure time. It is concluded that parents’ media literacy can improve preschool children’s quality of learning through democratic parenting, and there are group differences in mediating effects in different lengths of screen exposure time. Parents and educators should be aware of the impact of environments with media use on children.

## 1 Introduction

With the development of information technology, the importance of cybersecurity to individuals, companies, and countries has been increasingly significant. On September 22, 2023, the Organization for Economic Co-operation and Development (OECD) published *Building a Skilled Cyber Security Workforce in Latin America* (OECD, 2023), which advocates the importance of cybersecurity education. Cultivating a cybersecurity workforce has attracted international attention, and the content involved in the cultivation is closely related to media literacy. Research on cybersecurity education and awareness programs further highlights that successful initiatives often integrate media literacy components, such as misinformation recognition, privacy protection, and responsible digital practices (Zhang-Kennedy and Chiasson, 2021). For example, digital media literacy interventions have improved individuals’ ability to detect manipulated or deceptive content, a skill directly relevant to countering social engineering threats in cybersecurity contexts (Guess et al., 2020). Likewise, frameworks reconceptualizing cybersecurity awareness stress the importance of cognitive and behavioral capacities that overlap with media literacy dimensions (Akter et al., 2022). Systematic reviews of K–12 cybersecurity education also suggest that embedding media and digital literacy into curricula fosters long-term preparedness by cultivating critical thinking, security awareness, and resilience among future professionals (Livingstone et al., 2017). This body of evidence indicates that media literacy is not peripheral but central to developing a competent cybersecurity workforce. This reflects, to a certain extent, the important value in the information age. Due to the rapid development of media technology, emerging media featuring high portability and intelligence (such as smartphones and similar devices) have become prevalent in children’s lives and learning (Sitopu et al., 2024).

Given the prevalence of media in the lives of children, our attention turns to learning quality. Learning quality is a key capability for children to adapt to future development and engage in lifelong learning (Bustamante et al., 2017). Cultivating children’s quality of learning within the complex and ever-changing media environment is an important and noteworthy issue. Learning quality refers to all learning activities’ internal psychological tendencies and characteristics, including curiosity and interest, initiative, persistence, attention, creation and invention, reflection and explanation (Kagan et al., 1995). Learning quality refers not to specific knowledge and skills but to how children acquire and use knowledge and skills (Kagan et al., 1995; Washington State, 2004). It is closely related to children’s school preparations and high-quality academic level (Gijbels et al., 2005; Lee, 2012)—the early school period is an important period for developing children’s quality of learning. The development of their quality of learning is not only affected by internal factors such as temperament (Domínguez et al., 2011), age (Bell et al., 2013), but also external factors such as parents’ media usage habits (Sun, 2022) and parenting styles (Klebanov and Brooks-Gunn, 2008). A study by Lu (2022) found that the media use habits of both children and their parents directly influence the development of children’s quality of learning. Since modern individuals reside within the rituals and landscape of media culture (Mcluhan, 2001), parents and children must improve their media literacy, interpret media information accurately, and cultivate healthy media use habits.

The influence of parents’ media use behaviors and attitudes on preschool children’s learning and development has attracted attention from researchers (Radesky and Domoff, 2019; Nikken, 2017). However, the specific effect of parental media literacy on preschool children’s quality of learning is not yet apparent. Therefore, this study attempted to explore the mechanism of parental media literacy on preschool children’s quality of learning, and compared the mediating role of democratic parenting styles between parental media literacy and preschool children’s quality of learning in different lengths of screen exposure.

### 1.1 The relationship between media literacy and learning quality

Media literacy refers to individuals’ abilities to interpret and critically assess media information, as well as to utilize media information in relation to their personal life and social development, including the abilities to acquire, analyze, evaluate, create, and participate in media information (Zhang and Shen, 2004; Thoman and Jolls, 2008; Heiss et al., 2023). The media is a cognitive tool that helps people understand the world and is an important aspect of interpersonal interactions. Information has gradually become an important resource for human survival. Therefore, basic media literacy is essential to living in contemporary society. Since the family is a critical context for young children and their development and learning, parents’ media literacy plays a crucial role in children’s media literacy development.

According to social learning theory (Bandura and Walters, 1977), children acquire new behavioral responses and cognitive patterns by observing and imitating the behaviors, attitudes, and mental models of others—especially significant figures such as their parents. In the context of family media use, parents’ attitudes toward media, usage behaviors, and interaction styles serve as critical role models for children’s learning. When parents demonstrate a high level of media literacy—for example, critically evaluating media content, establishing appropriate usage rules, and engaging in joint media activities with their children—children are likely to internalize these behavioral patterns through the “observation–retention–reproduction” process (Rek and Kovačič, 2018). Parents guide children in understanding, evaluating, and responding to media information through explanations, emotional support, and co-participation in media activities (Namula and Wei, 2010; Smailova et al., 2023). Therefore, parental media literacy is not only a reflection of individual competence but also constitutes a key environmental factor in children’s development. This socialization process, rooted in observational learning, profoundly influences the formation of children’s learning quality.

Learning quality is an essential non-cognitive factor that permeates all learning activities and manifests as learners’ internal psychological characteristics and tendencies. According to the definition proposed by the National Education Goals Panel (NEGP), learning quality refers to children’s dispositions, attitudes, habits, and styles that reflect their ways of engaging in learning (Kagan et al., 1995). Specifically, it encompasses dimensions such as curiosity and interest, initiative, persistence and attention, imagination and creativity, and reflection and interpretation (Cai, 2015). Previous research has shown that parents with higher media literacy can cultivate a positive home media environment (Nathanson, 2015), foster children’s independent thinking, and encourage them to critically engage with information (Diergarten et al., 2017). Under such developmentally appropriate media guidance, children can develop critical thinking skills and exhibit higher levels of engagement in broader learning activities (Nikken and Schols, 2015; Fisch, 2014; Bukhalenkova et al., 2023). Ample empirical evidence supports this mechanism. For instance, some studies have found that parental media literacy has a greater impact on children’s information literacy than school-based education (Smailova et al., 2023), and it contributes to improved cognitive abilities and academic achievement (Pala and Basibuyuk, 2023; Kirkorian et al., 2008; Linebarger and Piotrowski, 2009). Moreover, parents’ approaches to organizing and presenting information can facilitate children’s knowledge transfer, enhance associative thinking, and stimulate creative potential (Bruner, 1978; Vygotsky and Cole, 1978). Their active engagement with digital media content and information filtering practices helps improve children’s attention and responsiveness (Barr et al., 2008) while promoting independent learning abilities (Taylor et al., 2024). By enhancing children’s media literacy, parents also help them construct effective models for receiving and screening information, strengthening their critical thinking skills (Diergarten et al., 2017). These studies collectively reveal a positive association between parental media literacy and children’s learning quality. Based on this, the present study proposes:

*H1*: Parental media literacy may have a significant positive impact on preschool children’s learning quality.

### 1.2 The mediating role of democratic education

Parenting style refers to the methods, attitudes, and behaviors parents adopt in educating and raising their children within the family environment. These behaviors are typically shaped by parents’ upbringing experiences, personal values, and media-related cognition, and are reflected in specific parenting practices (Huang, 2001). The democratic parenting style is characterized by high responsiveness and reasonable expectations. The democratic parenting style is characterized by high responsiveness and reasonable expectations. Democratic parenting style refers to a positive family environment that fosters democratic parent–child relationships. It is characterized by parents’ respect for children’s opinions, perspectives, and autonomy, as well as their tendency to be responsive to children’s needs (Bougher, 2018; Stattin et al., 2011; Wray-Lake and Flanagan, 2012). Democratic parenting style and democratic education share common philosophical roots, emphasizing respect, participation, and the development of autonomy. In family contexts, democratic parenting cultivates children’s ability to express opinions, engage in joint decision-making, and internalize values of fairness and reciprocity (Baumrind, 2013; Steinberg, 2001; Han and Yan, 2025; Ayık et al., 2025). These early socialization experiences provide the foundation for democratic education in school settings, where teachers create learning environments that encourage dialogue, cooperation, and critical thinking (Gutmann, 1993). Empirical studies have shown that students raised in democratic families are more likely to respond positively to participatory and dialogic pedagogical approaches, as they are already familiar with norms of voice, respect, and responsibility (Grolnick and Pomerantz, 2009). Thus, democratic parenting style at home can be regarded as a micro-level antecedent that supports and reinforces democratic education practices in formal schooling, forming a coherent developmental trajectory from family to school. It emphasizes providing children with ample respect and support while maintaining structured guidance. Parents with this style often guide their children’s behavior using analytical and encouraging approaches—for example, implementing reasonable reward and punishment systems, engaging in open dialogue, and encouraging independent thinking and expression. They strive to strike a dynamic balance between behavioral regulation and mutual respect, thus fostering a harmonious parent–child relationship (Reitman et al., 2002).

From the perspective of social learning theory (Bandura and Walters, 1977), children’s learning quality is not solely determined by intrinsic traits but is gradually shaped through observing and imitating parents’ behaviors in the home environment. Democratic parents serve as role models through positive communication, rational guidance, and emotional regulation. Through daily interactions, children observe how their parents approach problems, engage in reflection, and persist in their goals. They then internalize these behavioral patterns, developing corresponding cognitive styles and learning tendencies. This “observation–imitation–internalization” mechanism provides theoretical support for understanding how media literacy may influence children’s learning quality via parenting style.

Previous studies have shown that parental media literacy can influence their choice of parenting style. Parents with higher media literacy are more likely to adopt democratic parenting behaviors. When navigating the digital media environment, they tend to guide their children’s understanding of media content in an evidence-based manner, prioritize communication and negotiation, and respect their children’s interpretations and expressions of media messages (Livingstone et al., 2015; Özgür, 2016). For example, such parents are inclined to discuss the intent, authenticity, and value behind media content during co-use sessions, helping their children develop safe, rational, and healthy media habits (Zeng et al., 2020). This suggests that media literacy is reflected in parents’ media practices and profoundly shapes their everyday parenting strategies and interaction styles.

In child development research, the democratic parenting style is a crucial family factor in enhancing children’s learning quality. Studies have shown that democratic parents—through high-quality communication, autonomy support, and emotional encouragement—can foster higher levels of learning motivation, self-regulation, and reflective thinking in children. As a result, children tend to exhibit stronger learning qualities such as curiosity, attention, and creativity (Klebanov and Brooks-Gunn, 2008). Furthermore, respecting children’s viewpoints, especially during learning and exploration, is key in promoting deep learning tendencies and psychological engagement (Van Werkhoven et al., 2001).

Therefore, this study proposes Hypothesis 2: Democratic parenting style mediates the relationship between parental media literacy and preschool children’s learning quality.

### 1.3 Group differences in the screen exposure time of preschool children

Screen exposure refers to activities in which individuals are exposed to electronic media such as screens (Chen and Adler, 2019). Screen exposure time refers to how much time children spend on screen activities. The length of screen exposure time is related to the influence of media literacy on individuals. The longer children are exposed to screens, the more likely they are to be affected by parental media literacy (Mendoza, 2009; Terras and Ramsay, 2016).

The use-satisfaction model suggests that regulating children’s screen time is challenging once it has commenced (Hsu et al., 2015). When young children’s screen exposure exceeds 2 h, it can adversely affect their attentional flexibility, memory, learning abilities, and other cognitive functions (Pagani et al., 2010). Furthermore, there is a risk of adverse outcomes, such as academic difficulties, physical fatigue, and mental absorption (Zhang and Xu, 2021). Parents are advised to consciously analyze and screen the media information their children are exposed to. This process allows parents to strengthen the exchange of media information with their children, gain insight into their media thoughts and attitudes, enhance their cognitive attention, and can more significantly affect children’s learning ability (Griffith et al., 2022), consequently affecting their quality of learning. Studies have also pointed out that the higher the media literacy of parents is, the more scientific and reasonable the control and guidance of children’s e-product use time will be (Nikken and Schols, 2015), that is, the shorter the screen exposure time of children. This may indicate that the higher media literacy of parents plays a significant role in the learning quality of children (Hurwitz and Schmitt, 2020).

In addition, according to the time displacement hypothesis (Hussain, 2012), children who over-rely on and overuse electronic media have longer screen exposure time, which will affect the interaction between parents and children (Hong et al., 2019). In the process, children’s scientific control of screen exposure time can reduce the risk of over-dependence on the medium. Related research has shown that parents with low media literacy lack scientific management of children’s use of electronic media (Yadav and Chakraborty, 2022; Olszewski, 2015), with reduced frequency of dialogues with children (Pagani et al., 2010), and less democratic parenting (Huntington, 2016). This is not conducive to developing children’s attention (Meri et al., 2023) and critical thinking (Cladis, 2020). That is, children’s longer screen exposure time may mean that parents’ lower media literacy hurts children’s quality of learning. Therefore, hypothesis 3 is proposed in this study: In children with different lengths of screen exposure time, the influence of parental media literacy on democratic parenting and preschool children’s quality of learning may differ.

**Figure 1:**
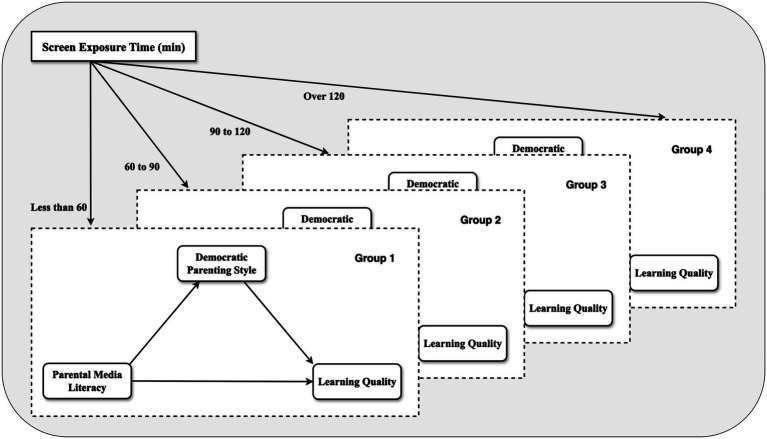
Theoretical model.

To sum up, this study intends to investigate the influence of parents’ media literacy on children’s learning quality, and further explore its mechanism—the mediating role of democratic parenting styles and the differences in groups of different lengths of screen exposure time, to provide an evidence-based foundation for enhancing parents’ media literacy and fostering young children’s learning quality. This will facilitate the creation of an optimal family media literacy environment, which is essential for promoting the healthy development of young children. Figure 1 shows the theoretical model of this study.

## 2 Methods

This study used SPSS macro PROCESS (Hayes, 2012) for descriptive statistics and correlation analysis. Bootstrap testing was used to assess the mediating effect, and AMOS 21.0 was used for multi-group analysis of the mediating model composed of parental media literacy, democratic parenting styles, and preschool children’s quality of learning.

### 2.1 Participants and procedure

To investigate the influence of parents’ media literacy on preschool children’s learning quality, this study collected and analyzed data from parents at four kindergartens in Hubei and Zhejiang provinces, China. These two provinces differ significantly in economic development and educational resources, thus providing a diverse representation of kindergarten education quality.

A cluster sampling method was employed. Four kindergartens (including both public and private institutions) were randomly selected from the official registry to ensure sample representativeness. All classes within each selected kindergarten were treated as sampling units, and all parents within those classes were invited to participate in the survey. The data collection process received strong support from kindergarten principals. Principals distributed the questionnaires to class teachers, who then shared the survey link and ethical statement with parents via parent WeChat groups. Teachers served only as third-party disseminators of the research information and did not influence or interfere with parents’ participation decisions. Parents completed the questionnaire online through the Wenjuanxing (WJX) platform, a widely used online survey tool in China known for its robust data privacy and security protections. The survey introduction prominently stated the study’s purpose and ethical guidelines, emphasizing voluntary participation and anonymity. Parents were informed that participation was entirely at their discretion and that their choice would not impact their child. All data were used solely for academic research and were collected directly by the researchers without access by kindergartens or related personnel.

Seven hundred nine questionnaires were distributed, and 603 valid responses were collected, resulting in a response rate of 85.05%. The questionnaires were answered mainly by mothers (473, 78.4%) and 115 fathers (115 19.1%), and the children related to the questionnaire consist of 302 boys (50.1%) and 301 girls (49.9%). About 0.8% of parents had completed school education, 11.1% had completed junior high school education, 27.5% had earned a senior high school or technical secondary education, 38.0% graduated from college, and 22.6% attended graduate school. Nearly 12.6% of parents were between the ages of 20–30, 68.3% were between the ages of 30–40, 17.1%of parents were between the ages of 40–50, and 2.0% were 50 years or older.

### 2.2 Tools

#### 2.2.1 Media literacy scale

The media literacy scale compiled by Qu (2020) was adopted to test the parents of preschool children. The scale consists of 32 questions and adopts a 5-level scoring method. The higher the score is, the higher the level of explanatory media literacy is. The scale is divided into four dimensions: basic media literacy, concept of media literacy education, attitude and method for media literacy education, and parent–child media interaction ability. (e.g., *When looking for information online, I will visit one or more websites and make a comprehensive comparison before deciding which information is worth referring to; I often try to find other ways to verify what is reported in newspapers, on TV, and on the Internet*). In this scale, the Cronbach’s *α* was 0.97, indicating excellent internal consistency.

#### 2.2.2 Learning quality scale

The parents of preschool children were tested with the “Parent Evaluation Scale for learning quality of Preschool Children” compiled by Cai (2015). The scale consists of 41 items and adopts the 4-level scoring method. The higher the score is, the higher the level of learning quality is. The scale is divided into five dimensions: curiosity and interest, initiative, persistence and attention, imagination and creation, and reflection and interpretation. (e.g., *Children can take the initiative to tell interesting things that happened in kindergarten; Children can stick to a good habit*). In this scale, the Cronbach’s α was 0.95, indicating excellent internal consistency.

#### 2.2.3 Parenting style scale

The questionnaire concerning parents’ styles compiled by Yang and Yang (1998) was used to test the parents of preschool children. There are 40 items in the scale. With the application of the 5-level scoring method, if the score of a dimension is high, it indicates that this type of parenting is more prominent. The scale is divided into five dimensions: drowning parenting style, democratic parenting style, dismissive parenting style, authoritarian parenting style, and inconsistent parenting style. Among them, the dimension related to the democratic parenting style includes items 8, 10, 12, 14, 17, 24, 27, 29, 33, and 37. (e.g., *Encourage your child to do what he knows how to do; Develop your child’s strengths according to their interests*). The Cronbach’s α of this scale and the dimension related to the democratic parenting style were 0.86 and 0.83, respectively, indicating good internal consistency.

#### 2.2.4 Screen exposure time

According to the definition of screen exposure time in relevant literature and the measurement of screen exposure time by Li and Liu (2022), this study investigated children’s screen exposure time in the fundamental information part of the questionnaire. The specific item was: “Children’s average daily exposure time to screen media,” with four response options: “less than 60 min, 60–90 min, 90–120 min, and more than 120 min.” To avoid boundary ambiguity, cases exactly at the boundary (e.g., 90 min) were coded into the upper-bound category (i.e., “90–120 min”).

## 3 Data analysis

### 3.1 Common method deviation test

The Harman single-factor test was applied for the standard method bias test, and exploratory factor analysis was conducted on all 113 items of media literacy, parenting style, and learning quality variables. The results showed that there were 22 factors with an eigenvalue greater than 1, and the variance explanation percentage of the first common factor was 23.25%, which is lower than the critical standard of 40%. Therefore, it can be considered that there is no serious common methodological bias in the data of this study.

### 3.2 Correlation analysis

The correlation analysis results of each variable show that parents’ media literacy significantly correlates positively with children’s learning quality and democratic parenting styles. Therefore, there is a significantly positive correlation between democratic parenting styles and children’s learning quality. The test results are shown in Table 1.

**Table 1:** Descriptive statistics and intercorrelations for all study variables.

Variable	*M*	*SD*	1	2	3
1. Media literacy	3.62	0.58	1		
2. Learning quality	2.87	0.44	0.538**	1	
3. Democratic parenting style	3.80	0.53	0.452**	0.463**	1

*p < 0.05, **p < 0.01, the same below.

### 3.3 Intermediate effect test

The results in Table 2 show that parents’ media literacy significantly affects children’s learning quality (*β* = 0.40, *p* < 0.01). After introducing mediating variables, parents’ media literacy has a significant direct effect on children’s learning quality (*β* = 0.31, *p* < 0.01), and it can significantly and positively predict democratic parenting styles (*β* = 0.49, *p* < 0.01). In addition, democratic parenting styles also significantly impacted children’s learning quality (*β* = 0.22, *p* < 0.01). It can be seen that a democratic parenting style plays the mediating role between parents’ media literacy and children’s learning quality.

**Table 2:** The relationship between media literacy and learning quality: an examination of the mediating effect of democratic parenting styles.

Regression equation		Overall fit index			Significance of the regression coefficient	
Result variable	Predictor	*R*	*R^2^*	*F*	*β*	*t*
Learning quality		0.55	0.30	52.78**		
	Parent’s age				0.02	0.71
	Parental monthly income				0.04	3.29**
	Media literacy				0.40	11.39**
Democratic parenting style		0.46	0.21	31.20**		
	Parent’s age				0.00	0.14
	Parental monthly income				0.04	2.52**
	Media literacy				0.49	13.98**
Learning quality		0.60	0.36	73.54**		
	Parent’s age				0.02	0.71
	Parental monthly income				0.03	2.56**
	Democratic parenting style				0.22	5.66**
	Media literacy				0.31	7.77**

*Indicates that the probability value obtained from the statistical test has reached the pre-set significance level.

### 3.4 Multi-group analysis of mediating models

In order to investigate the mediating effect of democratic parenting styles, media literacy, and learning quality on different lengths of screen exposure time, based on the above mediating model, a multi-group analysis was conducted using AMOS 21.0, and the screen exposure time was used as a group variable for testing. The fit analysis of the saturated and independent models found that the unrestricted model had the best fit, so the unrestricted model was selected as the multi-group analysis model. Based on the relationship between the variables and the research hypothesis, the mediating effect between media literacy, learning quality, and democratic parenting styles was investigated. AMOS21.0 was used to test the mediating effect, and the revised model results showed that the model fit index χ2df =2.80, RMSEA = 0.06, RMR = 0.02, GFI = 0.90, NFI = 0.92, CFI = 0.95, IFI = 0.95, NNFI = 0.93.

Based on verifying the good fit of the hypothesis model, the screen exposure time was taken as a group variable and divided into four periods, respectively, less than 60 min, 60–90 min, 90–120 min, and more than 120 min. Multi-group analysis was used to test the mediating effect of the hypothesis model on different groups. When the screen exposure time is less than 60 min, the three influence paths are significant, indicating that parental media literacy not only directly affects preschool children’s quality of learning, but also has an impact on preschool children’s quality of learning through democratic parenting styles; when the screen exposure time is 60–90 min, the influence of democratic parenting styles on the learning quality of preschool children is not significant; when the screen exposure time is 90–120 min, the influence of democratic parenting styles on the children’s quality of learning is not significant; when the screen exposure time is more than 120 min, the three influence paths are significant, indicating that parents’ media literacy not only directly affects preschool children’s quality of learning, but also has an impact on preschool children’s quality of learning through democratic parenting styles. The test results are shown in Table 3.

**Table 3:** Estimation results of the multi-group structural equation model.

Path	Screen exposure time of preschool children			
	Less than 60 min	60–90 min	90–120 min	Over 120 min
H1: Parental media literacy→Children’s quality of learning	0.226^***^	0.435^***^	0.282^***^	0.325^***^
H2: Parental media literacy→Democratic parenting style	0.387^***^	0.486^***^	0.282***	0.354^***^
H3: Democratic parenting style→Children’s quality of learning	0.332^***^	0.127	0.259	0.194^***^

*Indicates that the probability value obtained from the statistical test has reached the pre-set significance level.

## 4 Discussion

This study explored the impact of parental media literacy on preschool children’s learning quality in the context of new media, while also examining the mediating role of democratic parenting and group differences based on children’s screen exposure time. The results revealed that parental media literacy significantly positively affected children’s learning quality, with democratic parenting playing a significant mediating role in this relationship. Moreover, the mediation effect varied across groups with different levels of screen exposure. These findings highlight the importance of enhancing parents’ media literacy, adopting a democratic parenting style, and being more attentive to children’s screen time to support the development of their learning quality.

### 4.1 The positive impact of parental media literacy on children’s learning quality

This study is the first to systematically examine the influence of parental media literacy on preschool children’s learning quality. The findings revealed that parental media literacy significantly and positively predicted children’s learning quality. This result aligns with previous studies (Diergarten et al., 2017; Kirkorian et al., 2008; Linebarger and Piotrowski, 2009; Pala and Basibuyuk, 2023; Smailova et al., 2023; Taylor et al., 2024), and further provides both theoretical and empirical support for the relationship between parental media literacy and children’s learning quality.

This result also extends the applicability of social learning theory (Bandura and Walters, 1977) to the family context, highlighting the pivotal role parents play as media role models for young children. In early learning and daily life, parents’ media practices and interaction strategies provide observable behavior models and implicitly shape children’s patterns of information reception and processing. Parents with higher levels of media literacy are more capable of guiding children in a developmentally appropriate manner to filter, interpret, and critically engage with media content, thereby fostering more reflective and positive learning dispositions and abilities.

Moreover, parents’ demonstrated abilities in interpreting, evaluating, and analyzing media messages are essential socialization resources in shaping children’s media behaviors. Through observing and imitating these parents’ behaviors, children gradually internalize media strategies and value orientations, which in turn facilitate the development of their own information literacy and learning quality. Parents with high media literacy are also more likely to support their children with responsive and emotionally supportive interactions, stimulating their curiosity, learning interest, and critical thinking—ultimately enhancing their learning quality in attention, creativity, and reflective thinking.

### 4.2 The mediation effect of democratic education

This study also found that democratic parenting partially mediated the relationship between parental media literacy and preschool children’s learning quality. This finding echoes previous research (Livingstone et al., 2015; Özgür, 2016; Zeng et al., 2020) and further provides theoretical and empirical support for the role of democratic parenting in linking parental media literacy to children’s learning outcomes.

This result expands the theoretical understanding of the “media literacy–learning quality” pathway and provides strong empirical support for social learning theory (Bandura and Walters, 1977). According to this theory, children construct their cognitive and behavioral patterns by observing significant others—particularly their parents—in everyday situations, including their behavioral expressions, communication styles, and emotional regulation. In this study, parental media literacy manifests not only in information processing skills but also in how parents interact with their children—specifically, in their communication, guidance, and behavioral norms embodied in their parenting style.

Parents with higher levels of media literacy are more likely to adopt a democratic parenting style, suggesting that media literacy is not merely a technical or cognitive competence but also a dispositional quality embedded in everyday parenting practices (Meng et al., 2021). Specifically, such parents are more inclined to respond to their children’s media behaviors with analytical guidance and respect for the child’s perspective—for example, by offering moderate choice, engaging in empathetic communication, and explaining the meaning and potential risks of media content (Özgür, 2016; Zeng et al., 2020). These interactive practices provide observable and imitable behavioral models for children and foster learning-related dispositions such as curiosity, critical thinking, and reflective ability, enhancing overall learning quality (Ye et al., 2021).

From a theoretical perspective, parental media literacy indirectly influences children’s learning quality through an externalized parenting behavior pathway. This finding reinforces the core mechanism of social learning theory—the chain of modeling, imitation, and internalization. High parents’ media literacy promotes children’s information literacy through behavioral modeling and shapes a supportive learning environment through parenting practices. This mechanism supports an important extension of social learning theory: children imitate behaviors and observe and internalize the underlying values and interaction patterns embodied in those behaviors.

### 4.3 The impact of children’s different screen exposure times

This study further revealed that the effects of the mediation model pathways differed significantly across screen exposure time conditions. Specifically, when children’s daily screen exposure was less than 60 min or more than 120 min, democratic parenting significantly mediated the relationship between parental media literacy and preschoolers’ learning quality. However, this mediation effect was insignificant within the 60–120-min exposure range. This pattern of results indicates that the strength of the mediation model varies across different levels of children’s screen exposure, rather than following a simple linear trend. Such group-based differences are consistent with previous findings (Kerai et al., 2022; Sticca et al., 2025), offering empirical support for identifying optimal time windows for parents’ intervention and highlighting the pivotal role of highly media-literate parents in effectively mediating children’s learning outcomes under either very short or very long screen exposure conditions (Xie et al., 2024).

Several theoretical and empirical perspectives can explain this variation in the mediation pathway. Possible reasons include that the displacement hypothesis posits that screen time may crowd out opportunities for face-to-face interactions with significant others (e.g., parents or peers), thus diminishing the impact of parenting styles—such as democratic parenting—that rely heavily on interpersonal interaction (Hussain, 2012; Sticca et al., 2025; Putnick et al., 2023). When children are exposed to screens less than 60 min per day, parent–child interaction remains ample, allowing parents to naturally implement democratic practices—such as explanation, guidance, and empathetic dialogue—that positively impact learning quality. On the other hand, when children’s daily screen exposure exceeds 120 min, the media content they encounter becomes increasingly complex, fragmented, and information-dense, heightening the risk of the displacement effect associated with digital media use. Parents with higher levels of media literacy tend to adopt democratic parenting strategies to buffer the potential risks of prolonged exposure. Rather than simply prohibiting or indulging their children’s screen use, they engage in negotiation and guided mediation, transforming part of the screen time into opportunities for shared parent–child learning. Through such practices, parents create a supportive and controllable environment that ensures children can use media in a healthy and developmentally appropriate manner. In such cases, parents with high media literacy are more likely to engage in structured yet open forms of mediation, including democratic communication strategies, to buffer the adverse effects of excessive media exposure. The functionality of democratic parenting is thus reactivated in this context.

Furthermore, parental media literacy determines how digital information is processed and shapes cognitive tendencies in parenting strategies. Research shows that media-literate parents tend to employ explanatory, strategic, and empathetic approaches when navigating complex media environments—such as high screen exposure—which in turn support children’s information filtering, cognitive processing, and self-regulation skills (Griffith et al., 2022; Xie et al., 2024).

Interestingly, within the moderate screen exposure group (60–120 min), the mediating role of democratic parenting was not significant. The displacement hypothesis does not simply assume that “more media use is inherently harmful,” but rather emphasizes that media use tends to replace other developmentally beneficial activities, thereby influencing children’s learning and cognitive growth. Moreover, the effects of media exposure are not linearly increasing but instead display a threshold-like pattern. Parents may perceive one to 2 h of daily screen exposure as a manageable and safe duration that does not substantially interfere with their children’s other developmental activities. Consequently, they adopt a low-intervention approach, allowing relatively free media use without displaying clear democratic parenting behaviors. Compared with other exposure groups, this finding reveals what might be considered the perceived safe range of screen use among Chinese parents. When screen exposure is short (within 60 min), parents feel little concern and can fully exercise a democratic parenting style. When exposure reaches a moderate level (60–120 min), it falls into parents’ perceived “safe zone.” However, when exposure exceeds 2 h per day, parents become concerned and tend to actively intervene through democratic parenting behaviors as a form of protective regulation.

These findings also reveal a behavioral blind spot and lagging regulatory mechanism among Chinese parents when facing moderate-to-high screen exposure contexts. Kerai et al. (2022), drawing on large-scale data from Canadian preschoolers, noted that daily screen time exceeding 1 h significantly increased children’s vulnerability in cognitive, social, emotional, and language development domains. This highlights the need for future studies to explore screen exposure effects’ boundary conditions and ecological contextual variables—especially in China’s ongoing digital parenting transformation. Moreover, cross-cultural comparative research is warranted to examine how the interactions among parental media literacy, parenting styles, and children’s learning quality may vary across different societal structures and media-use cultures.

## 5 Conclusion

To sum up, this study demonstrates that within the mediating model, which encompasses parents’ media literacy, children’s learning quality, and democratic parenting styles, there are several significant positive predicting relationships: parents’ media literacy can positively predict children’s learning quality, parents’ media literacy can positively predict democratic parenting styles, and democratic parenting styles can positively predict children’s learning quality. In addition, the aforementioned mediating model is also affected by the screen exposure time of children, and the mediating model has different influence paths in groups with different lengths of screen exposure time. It is clear that the ‘information age’ is shaping the lives of children. Learning and thinking in the online world woven by online technology is a considerable challenge for media literacy education. Within the family—the central environment for young children’s media interaction—parental media literacy is indispensable in developmental trajectories. This study found that parental media literacy and democratic parenting styles had a significantly positive correlation with children’s quality of learning. Therefore, on one hand, parents should improve their own level of media literacy, critically selecting and filtering information content in a media society of information overload to choose information that is healthy and aligns with the physical and mental development of children, and to play a supervisory and corrective role in the process of children’s exposure to and absorption of media information. This implies an urgent need for parents to improve their media literacy. Consequently, parents must set a good example, strictly regulate their media usage habits, and strengthen their children’s joint use of media. In this process, they should guide their children to access appropriate information for their age and stage. As parents serve as role models for their children, they must provide guidance and support when their children engage with the outside world, safeguarding them from the influence of undesirable or harmful information.

On the other hand, it was found that parental media literacy can positively influence democratic parenting styles, implying that parents with high media literacy tend to adopt democratic parenting styles, conducive to improving young children’s media literacy. Previous research has shown that parental media literacy can facilitate the development of children’s media literacy skills (Lee and Kwon, 2024), and democratic parenting styles are significantly and positively related to children’s media literacy (Yusuf et al., 2020). Therefore, the adoption of democratic parenting styles by parents with high media literacy has the potential to contribute to improving children’s media literacy. Parents should also adopt a democratic parenting style, sharing and exchanging information content with their children through an understanding, respectful, democratic and equal approach to parenting attitude and dialogue, thus improving their children’s media literacy, satisfy and stimulate their curiosity in processing information, enhance their initiative in learning, and develop their creative thinking, thereby promoting the development of their learning qualities.

Parents should help children to satisfy and stimulate their curiosity in the process of information processing, enhance their learning initiative, and develop their creative thinking, to promote the development of children’s learning. Last, parents should reasonably control children’s screen exposure time. Parents with democratic parenting styles tend to adopt the media communication strategy of equal dialogue, patiently listen to their children’s needs, effectively help their children select useful media information, and provide them with techniques and methods for media engagement. This approach aims to enhance children’s media use efficiency and reduce the harm caused by excessive screen exposure. As important role models, parents should set an example to control screen exposure time rationally. Moreover, parents should screen the content their children are exposed to, opting for educational, engaging, and interactive material. Parents can create a more harmonious family environment by strengthening joint media use with their children, enhancing communication about media content, and improving the quality of their companionship.

### 5.1 Strengths and limitations

This study contributes to the mechanism of parental media literacy on the quality of learning of preschool children. It reveals the direct predictive effect of parental media literacy on preschool children’s quality of learning and the mediating effect of democratic parenting styles, and it also points out the group differences in different lengths of screen exposure time.

This study has several limitations. Firstly, the participants were parents of children in only four kindergartens in southern China. The generalizability of the findings is limited and can be enhanced through a systematic approach to enroll more parents with different socioeconomic backgrounds. Although the sample covered kindergartens of different types (public and private) and from two provinces that vary considerably in economic and educational development, it primarily represented urban and suburban contexts. The exclusion of rural kindergartens may limit the generalizability of the findings, as family media literacy and educational engagement may differ substantially in rural areas. Future research should therefore expand sampling to rural regions to obtain a more comprehensive picture of the relationships examined in this study. Secondly, this study is mainly based on a cross-sectional design, and the method is relatively simple; thus, it is difficult to infer causality from the research results. In future studies, longitudinal design, qualitative research, and other methods can also be applied to analyze the causal relationship between parents’ media literacy and children’s learning quality. The development of children’s media literacy and learning quality cannot be separated from the mutual coordination and cooperation of the family, kindergarten, and community. Future research should investigate collaborative frameworks and communication channels among families, kindergartens, and communities to establish effective cooperation mechanisms that enhance children’s adaptive capacities in the rapidly evolving new media era. In addition, some potential confounding factors should be noted when interpreting the conclusions of this study. For instance, parents’ educational attainment and the home media environment may influence both their likelihood of adopting a democratic parenting style and their children’s learning quality. These factors were not fully controlled for in the present analysis due to data limitations. Future research should take these important covariates into account to provide a more comprehensive understanding of the mechanisms underlying the associations observed in this study.

## Data Availability

The raw data supporting the conclusions of this article will be made available by the authors, without undue reservation.
